# T2WI-based MRI radiomics for the prediction of preoperative extranodal extension and prognosis in resectable rectal cancer

**DOI:** 10.1186/s13244-024-01625-8

**Published:** 2024-02-27

**Authors:** Hang Li, Li Chai, Hong Pu, Long-lin Yin, Mou Li, Xin Zhang, Yi-sha Liu, Ming-hui Pang, Tao Lu

**Affiliations:** 1 Department of Radiology, Sichuan Provincial People’s Hospital, University of Electronic Science and Technology of China, 32# Second Section of First Ring Road, Qingyang District, Chengdu, Sichuan 610070 China; 2 Beijing Tiantan Hospital, Capital Medical University, Beijing, China; 3 Institute of Radiation Medicine, Sichuan Provincial People’s Hospital, University of Electronic Science and Technology of China, Chengdu, China; 4 Pharmaceutical Diagnostic Team, GE Healthcare, Beijing, 100176 China; 5 Department of Pathology, Sichuan Provincial People’s Hospital, University of Electronic Science and Technology of China, 32# Second Section of First Ring Road, Qingyang District, Chengdu, Sichuan 610070 China; 6 Department of Geriatric Surgery, Sichuan Provincial People’s Hospital, University of Electronic Science and Technology of China, 32# Second Section of First Ring Road, Qingyang District, Chengdu, Sichuan 610070 China

**Keywords:** Lymph node, Rectal Neoplasms, Magnetic resonance imaging, Nomograms

## Abstract

**Objective:**

To investigate whether T2-weighted imaging (T2WI)-based intratumoral and peritumoral radiomics can predict extranodal extension (ENE) and prognosis in patients with resectable rectal cancer.

**Methods:**

One hundred sixty-seven patients with resectable rectal cancer including T3T4N + cases were prospectively included. Radiomics features were extracted from intratumoral, peritumoral 3 mm, and peritumoral-mesorectal fat on T2WI images. Least absolute shrinkage and selection operator regression were used for feature selection. A radiomics signature score (Radscore) was built with logistic regression analysis. The area under the receiver operating characteristic curve (AUC) was used to evaluate the performance of each Radscore. A clinical-radiomics nomogram was constructed by the most predictive radiomics signature and clinical risk factors. A prognostic model was constructed by Cox regression analysis to identify 3-year recurrence-free survival (RFS).

**Results:**

Age, cT stage, and lymph node-irregular border and/or adjacent fat invasion were identified as independent clinical risk factors to construct a clinical model. The nomogram incorporating intratumoral and peritumoral 3 mm Radscore and independent clinical risk factors achieved a better AUC than the clinical model in the training (0.799 vs. 0.736) and validation cohorts (0.723 vs. 0.667). Nomogram-based ENE (hazard ratio [HR] = 2.625, 95% CI = 1.233–5.586, *p* = 0.012) and extramural vascular invasion (EMVI) (HR = 2.523, 95% CI = 1.247–5.106, *p* = 0.010) were independent risk factors for predicting 3-year RFS. The prognostic model constructed by these two indicators showed good performance for predicting 3-year RFS in the training (AUC = 0.761) and validation cohorts (AUC = 0.710).

**Conclusion:**

The nomogram incorporating intratumoral and peritumoral 3 mm Radscore and clinical risk factors could predict preoperative ENE. Combining nomogram-based ENE and MRI-reported EMVI may be useful in predicting 3-year RFS.

**Critical relevance statement:**

A clinical-radiomics nomogram could help preoperative predict ENE, and a prognostic model constructed by the nomogram-based ENE and MRI-reported EMVI could predict 3-year RFS in patients with resectable rectal cancer.

**Key points:**

• Intratumoral and peritumoral 3 mm Radscore showed the most capability for predicting ENE.

• Clinical-radiomics nomogram achieved the best predictive performance for predicting ENE.

• Combining clinical-radiomics based-ENE and EMVI showed good performance for 3-year RFS.

**Graphical Abstract:**

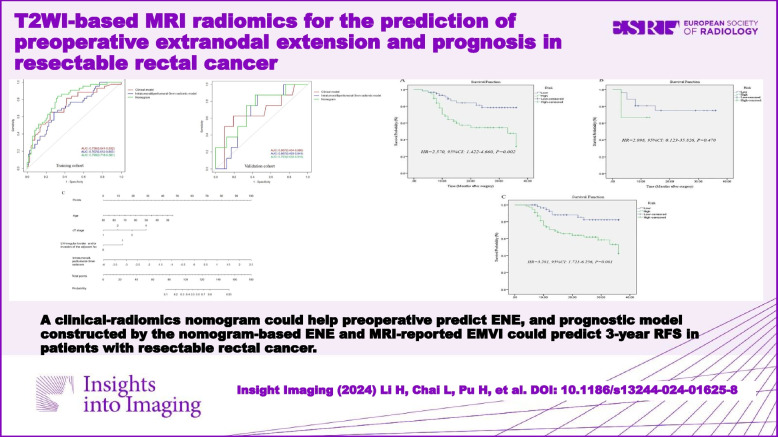

**Supplementary Information:**

The online version contains supplementary material available at 10.1186/s13244-024-01625-8.

## Introduction

Colorectal cancer ranks as the second most common cause of cancer mortality in the world [1]. Although most patients with resectable rectal cancer undergo curative resection, distant recurrence is the main cause of rectal cancer death [2]. Therefore, it is important to accurately assess risk factors for recurrence to improve survival. Extranodal extension (ENE) is the breakthrough growth of tumor cells from within the lymph node (LN) capsule into the surrounding perinodal adipose tissues [3]. Given this, ENE is known to be of important prognostic value in a variety of malignant tumors [4–7]. ENE has been recently incorporated into LN staging in neck cancer [8]. In rectal cancer, several previous studies indicated that ENE was an adverse factor for recurrence-free survival (RFS) [9–11]. Moreover, the panel agrees that radiologists should no longer consider the mesorectal fascia as involved when potentially malignant smooth enlarged lymph nodes (i.e., with an apparently intact capsule) contact the mesorectal fascia [12]. Therefore, these studies suggest that the preoperative prediction of ENE is an important parameter in reflecting the risk of recurrence and determining adjuvant treatment strategies [9–11].

Traditional imaging methods that depend on qualitative evaluation alone cannot reliably identify ENE in neck cancer, with an area under the receiver operating characteristic (ROC) curve (AUC) of 0.621–0.700 [13, 14]. Moreover, the size of node metastasis in head and neck squamous cell carcinoma is usually larger than that in rectal cancer [15]. Therefore, we hypothesized that the preoperative evaluation of ENE in patients with rectal cancer using qualitative evaluation alone was also not reliable. At present, no study has been performed to assess ENE in patients with rectal cancer using a method based on radiomics. Previous studies showed that the MRI-based radiomics signature score (Radscore) of the primary tumor could be used to identify lymph node metastasis (LNM) and tumor deposits in rectal cancer [16, 17]. However, most of these studies only focused on intratumoral regions, while peritumoral regions, which may have important information about the tumor, were excluded [18–20]. Heterogeneity exists not only in cancer cells but also in nonmalignant cells and infiltrating cells around the tumor, usually referred to as the peritumoral microenvironment [21]. Tumor evolution and progression are influenced by the interaction between cancer cells and the peritumoral microenvironment [22]. To our knowledge, no study has been performed to investigate the relationships between preoperative MRI-Radscore-based ENE and 3-year RFS. The Radscore from T2-weighted imaging (T2WI) of the primary tumor alone has been reported to be useful for predicting the response to chemoradiotherapy in rectal cancer [23]. Therefore, the primary aim of this study was to develop and validate a radiomics approach for the preoperative prediction of ENE based on intratumoral and peritumoral tissue on T2WI images in patients with rectal cancer undergoing radical resection. The secondary aim was to evaluate whether this predictive model-based ENE was associated with 3-year RFS in patients with rectal cancer.

## Materials and methods

### Patients

This prospective study was approved by the institutional review board of our hospital, and written informed consent was obtained from all patients. The study complied with the Declaration of Helsinki.

**Fig. 1:**
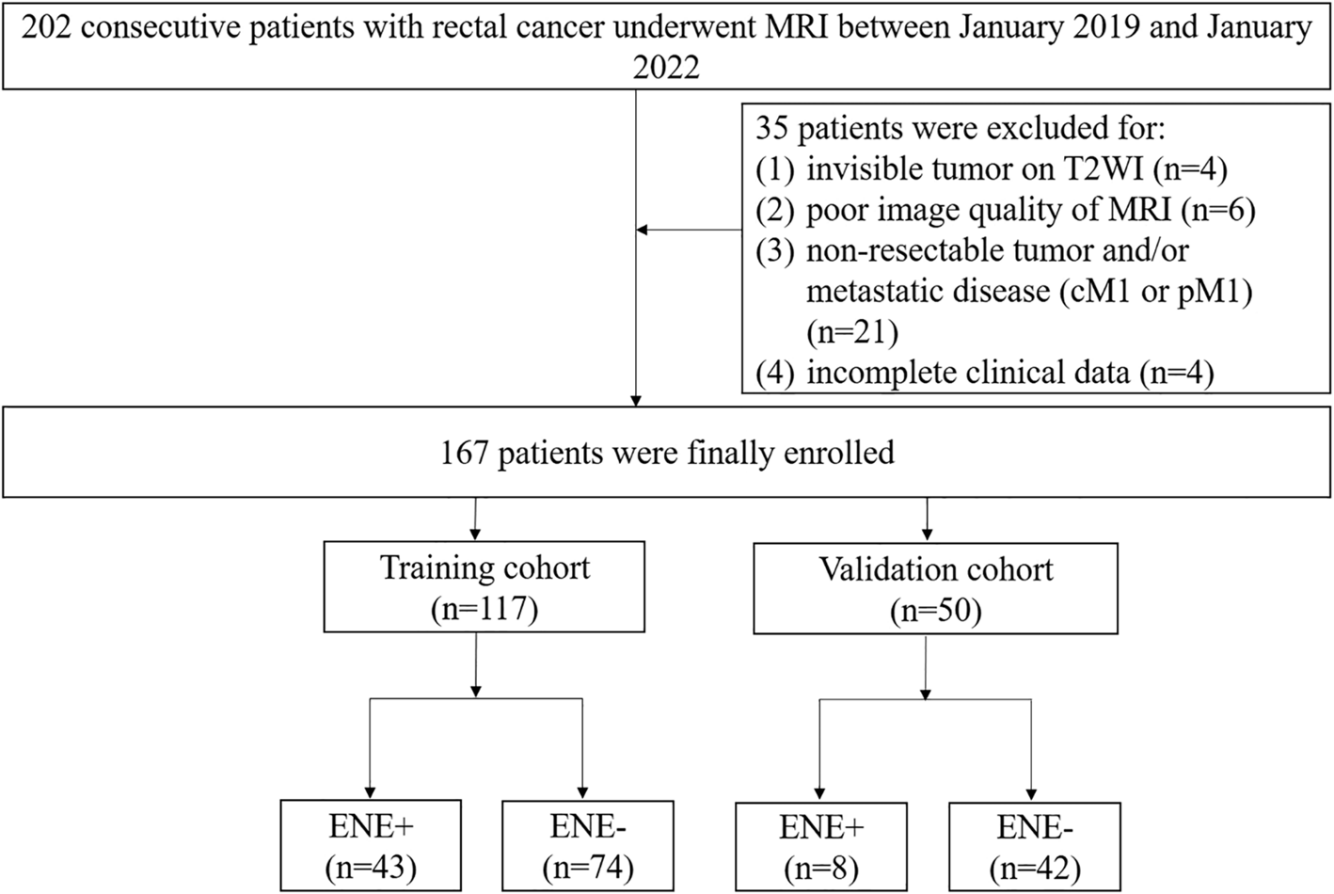
Flowchart of patient selection

From January 2019 to January 2022, 202 patients with rectal cancer deemed resectable based on the results of preoperative MRI were enrolled. Total mesorectal excision was performed in all patients. The inclusion criteria were as follows: (1) patients who received radical surgery without preoperative adjuvant therapy, (2) rectal cancer and LN status confirmed by pathological results, and (3) complete high-resolution rectal MRI examination data recorded 2 weeks before surgery. The exclusion criteria were as follows: (1) tumor invisible on T2WI images (*n* = 4); (2) poor MRI image quality (*n* = 6); (3) nonresectable tumor and/or metastatic disease (cM1 or pM1) (*n* = 21); and (4) incomplete clinical data (*n* = 4). Ultimately, 167 patients were included in this study. Among the 167 patients, there were 60 patients without lymph node metastasis (LNM), 29 patients with 1 LNM, 47 patients with 2–3 LNMs, 22 patients with 4**–**6 LNMs, and 9 patients with 7 or more LNMs. According to the eighth edition American Joint Committee on Cancer (AJCC) rectal cancer staging system [8], there were 60 patients at stage pN0, 29 patients at stage pN1a, 47 patients at stage pN1b, 22 patients at stage pN2a, and 9 patients at stage pN2b. Baseline characteristics of the patients, including carbohydrate antigen 199 (CA199), carcinoembryonic antigen (CEA), sex, and tumor location, were also recorded. There were 117 patients in the training cohort and 50 patients in the validation cohort. A flowchart of the study participants is described in Fig. 1.

### Imaging protocol

MRI was performed on a 1.5-T MR scanner (MAGNETOM Aera, Siemens Healthineers). Scopolamine butylbromide (20 mg) (Buscopan, Boehringer Ingelheim) was intramuscularly injected to inhibit bowel motion 10 min before the MRI examination. Water or air was not given to dilate the rectum. Axial T2WI without fat saturation was performed, and the scanning direction was oriented perpendicular to the long axis of the rectum. The following parameters were used: TR, 4600; TE, 75; field of view (FOV), 220 mm^2^; matrix size, 256 × 512; and 3 mm thickness with no interslice gap. Diffusion-weighted imaging parameters were as follows: TR, 4600; TE, 59; number of signals acquired, eight; FOV, 360 mm^2^; 5 mm section thickness; and *b* = 0, 800 s/mm^2^.

### Qualitative image evaluation

Two radiologists (with 5 and 12 years of experience in rectal cancer) who were blinded to the clinical information reviewed the MR images in 167 patients with rectal cancer. Radiologic ENE was considered positive when at least one of the following criteria was met: (1) irregular LN border and (2) invasion of the adjacent fat [13]. Additionally, we assessed whether the internal intensity of LN was heterogeneous and whether the chemical shift effect (CSE) along the margin of LN was absent [24]. Tumor cells inside the subcapsular sinus that break through the LN capsule may influence the uniform fat-water interface and thus destroy the normal CSE. If the LN capsule is broken by the tumor cell, the normal chemical shift effect in this region will disappear. The heterogeneous intensity of the LN indicates that the normal LN structure is replaced by tumor cells, and the low-intensity signal of the LN capsule on T2WI will disappear. Radiologic ENE status was compared with the nodal histopathology results (Fig. S1). For a node-to-node comparison, the following morphological features were recorded: LN size and the location related to the tumor, mesorectal fascia, and vessels. If the LNs on MRI did not match the histopathological results, these LNs were excluded. Finally, the pathological findings determine whether LNM and ENE are positive or negative. The LN yield at pathology per patient was more than 12. The extramural vascular invasion (EMVI) status of the primary tumor and T stage were also evaluated on MRI [25]. Moreover, the tumor length and maximal tumor thickness were obtained on the sagittal and oblique T2WI images, respectively.

### Tumor segmentation

**Fig. 2:**
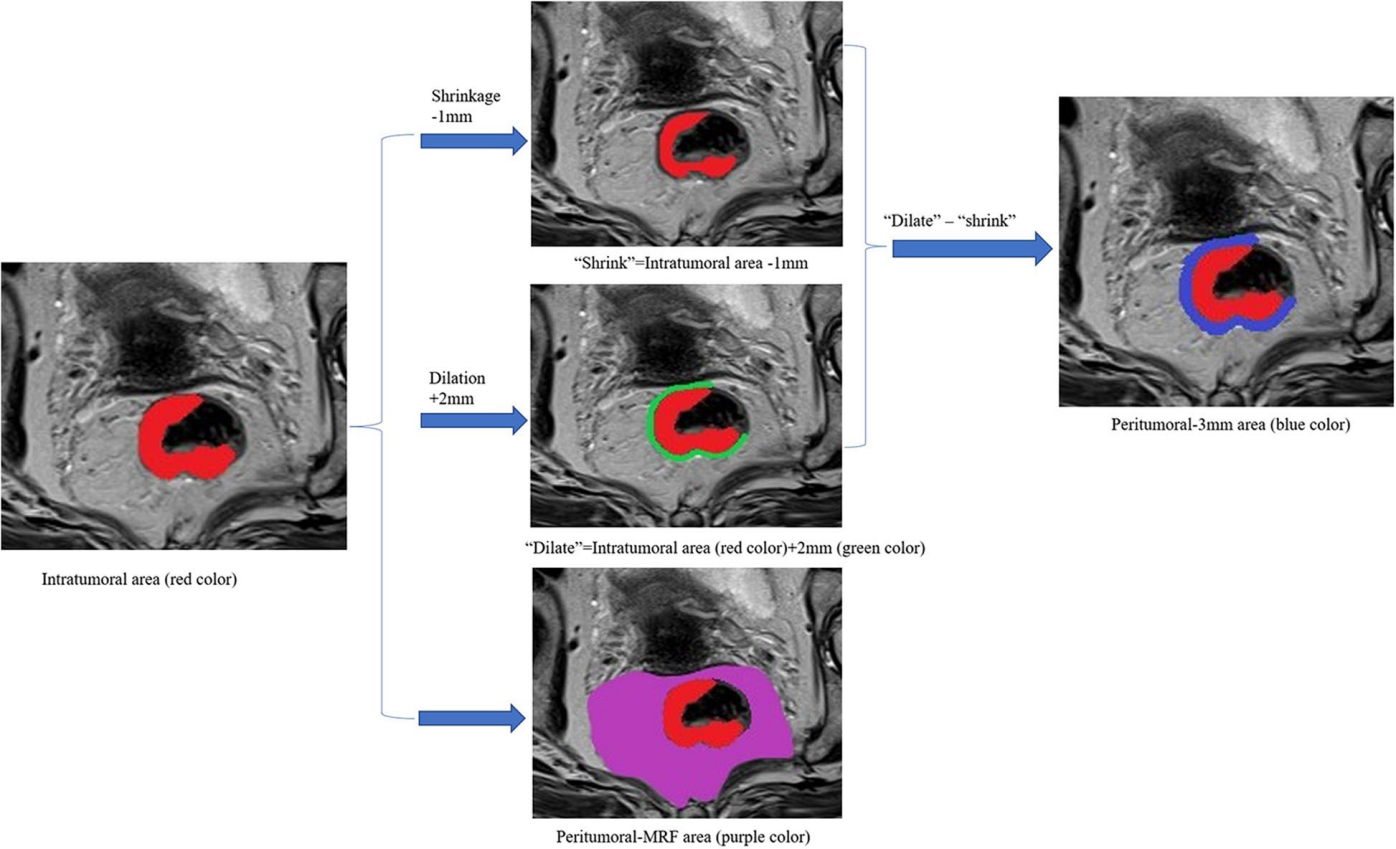
Tumor segmentation process on MRI. First, we manually segmented the whole tumor on axial T2WI images and labeled it as the intratumoral area. Second, “shrink” was defined as the tumor border automatically shrinking by 1 mm on the inside. “Dilate” was defined as automated dilation of the tumor border by 2 mm on the outside. “Dilate-shrink” resulted in a ring with a thickness of 3 mm. Thus, the peritumoral 3 mm area was obtained, including the most peripheral portion of the tumor and the surrounding tissues. Moreover, the peritumoral-mesorectal fat (MRF) area was obtained by drawing along the mesorectal fascia

The tumor segmentation process on MRI is shown in Fig. 2. Tumor delineation was performed on the entire three-dimensional tumor volume on T2WI images by a radiologist (the first author) using AK software (Artificial Intelligence Kit, version 3.3.0, GE Healthcare). For the segmentation of peritumoral regions, a peritumoral 3 mm region was obtained with automated dilation of the tumor boundaries by 2 mm on the outside and shrinkage of the tumor boundaries by 1 mm on the inside, resulting in a ring with a thickness of 3 mm [26]. The peritumoral-mesorectal fat (MRF) region was obtained by drawing along the mesorectal fascia. Thirty patients’ data were randomly selected for assessing interobserver and intraobserver agreement of feature extraction by intraclass correlation coefficient (ICC) analysis. First, a radiologist delineated the tumor volume of interest on T2WI images and repeated this process after 3 weeks to calculate the intraobserver ICC. Two radiologists independently delineated the tumor volume of interest on T2WI images, and the interobserver ICC was calculated. Only radiomics features demonstrating an ICC greater than 0.75 were retained for further analysis.

### Feature extraction, selection, and model building

The radiomics module (backed by PyRadiomics) was used to extract radiomics features. Voxel size was resampled by 1 × 1 × 1 mm, and *z* score normalization of the signal intensities for T2WI images was performed using PyRadiomics [27]. In total, 1316 radiomics features, including 252 histogram features, 14 shape features, 336 Gy level co-occurrence matrix, 224 Gy level size zone matrix, 224 Gy level run length matrix, 196 Gy level dependence matrix, and 70 neighboring gray-tone difference matrix, were obtained from each patient. The statistical software R (version 3.5.1, 2019, The R Foundation for Statistical Computing, Vienna, Austria) was used to select radiomics features and build the model. The maximum relevance and minimum redundancy were first performed to select radiomics features [28]. The optimized subset of features was obtained using the least absolute shrinkage and selection operator in the training cohort [29]. Subsequently, a linear combination of the selected features weighted by their respective coefficients was used to calculate the Radscore for each patient. The diagnostic performance of the Radscore in the training cohort and validation cohort was evaluated using AUC. The most predictive Radscore was selected for the subsequent analysis.

The nomogram was constructed by univariate and multivariate logistic regression analyses. ROC curves were generated to assess the discriminatory ability in the training cohort and validation cohort. A calibration curve was generated to calibrate the nomogram. Decision curve analysis (DCA) was applied to investigate the clinical utility of the models.

### Outcome

For patients with postoperative T1-2N0M0 rectal cancer, only the “follow-up watch” strategy was used. Patients with postoperative T3a/bN0M0, T4aN0M0, or T1-4aN1-2M0 rectal cancer received 5-fluorouracil-based adjuvant therapy after surgery. Locoregional recurrence or distant metastasis after surgery was evaluated every 3–6 months based on digital rectal examination and endoscopic examination plus CT, MRI, and/or PET/CT to determine relapse. The primary endpoint was a 3-year RFS.

### Statistical analysis

Statistical analyses were performed using SPSS (version 23.0) and R software (version 3.5.1). An interreader agreement was conducted for the assessment results of ENE by the two radiologists using the kappa value. The relationship between clinical baseline characteristics and ENE status was evaluated by the chi-squared test, independent two-sample *t* test, and Fisher’s exact test (where appropriate). The “mRMR” algorithm in the “mRMRe” package was used to conduct the maximum relevant minimum redundancy to initially screen the radiomics features. The best feature cohort was selected by the “glmnet” algorithm in the “glmnet” package. ROC analysis was carried out based on the “pROC” package to evaluate the effectiveness. The “calibrate” function in the “rms” package was applied to calibration curve plots and builds nomograms, and decision curves were plotted based on the “rmda” package in both clinical and combined models. The differences in AUCs between the models were compared using Delong’s test. Kaplan–Meier analysis with the log-rank test was used for survival analysis. Univariate and multivariate Cox regression analyses were used to construct a prognostic model for assessing 3-year RFS. The diagnostic performance of this prognostic model was determined using time-dependent ROC curves.

## Results

### Patient characteristics

Among the 167 patients (mean age, 62 years; range 29–88 years), 117 patients were in the training cohort (43 ENE + and 74 ENE-), and 50 patients were in the validation cohort (8 ENE + and 42 ENE-). There was a significant difference in pathological ENE between the two cohorts (*p* = 0.008), but no significant differences were found in CA199, CEA, tumor length, wall thickness, age, sex, location, cT stage, pathological LN, MRI-reported EMVI, LN-irregular border and/or adjacent fat invasion, LN-CSE, and LN-heterogeneous intensity between the two cohorts (all *p* > 0.05) (Table 1).

**Table 1:** Baseline characteristics of the patients in this study

Variable	Training cohort (*n* = 117)	Test cohort (*n* = 50)	*p*-value
CA199 (kU/L)	7.85 (4.05, 17.86)	8.76 (3.77, 17.63)	0.760
CEA (ng/mL)	4.43 (2.61, 10.41)	3.40 (2.16, 8.10)	0.259
Tumor length (mm)	45.00 (34.50, 57.00)	46.65 (36.75, 60.75)	0.483
Maximal tumor thickness (mm)	10.00 (8.00, 13.00)	10 (7.00, 12.00)	0.232
Age	64.00 (55.00, 69.50)	67.50 (56.75, 71.00)	0.369
Sex			0.375
Male	83 (70.21%)	32 (72.73%)	
Female	34 (29.79%)	18 (27.27%)	
Location			0.521
Upper	23 (28.72%)	13 (27.27%)	
Middle	60 (44.68%)	26 (63.64%)	
Lower	34 (26.60%)	11 (9.09%)	
cT stage			0.557
T1	1 (4.26%)	1 (0.00%)	
T2	21 (47.87%)	6 (50.00%)	
T3a/b	41 (17.02%)	21 (31.82%)	
T4a	54 (30.85%)	22 (18.18%)	
pN stage			0.206
N0	39 (33.33%)	21 (42%)	
N1a	18 (15.38%)	11 (22%)	
N1b	33 (28.21%)	14 (28%)	
N2a	19 (16.24%)	3 (6%)	
N2b	8 (6.84%)	1 (2%)	
Pathological LN			0.285
Negative	39 (33.33%)	21 (42%)	
Positive	78 (66.67%)	29 (58%)	
Pathological ENE			0.008
Negative	74 (63.3%)	42 (84%)	
Positive	43 (36.7%)	8 (16%)	
MRI-reported EMVI			0.321
Negative	93 (69.15%)	43 (77.27%)	
Positive	24 (30.85%)	7 (22.73%)	
LN-irregular border and/or adjacent fat invasion			0.990
Negative	75 (64.10%)	32 (64.00%)	
Positive	42 (35.90%)	18 (36.00%)	
LN-CSE			0.985
Absence	63 (53.85%)	27 (54.00%)	
Existence	54 (46.15%)	23 (46.00%)	
LN-heterogeneous intensity			0.598
Negative	77 (65.81%)	35 (70.00%)	
Positive	40 (34.19%)	15 (30.00%)	

*CA199* carbohydrate antigen 199, *CEA* carcinoembryonic antigen, *LN* lymph node, *CSE* chemical shift effect, *EMVI* extramural vascular invasion

### MR-reported ENE correlation with pathologic results

The correlation of MR-reported ENE and pathologic results is shown in Table S1.

Pathologically confirmed ENE positivity was observed in 51 patients, with an MR-reported ENE positivity in 27 patients for reader 1 and 26 patients for reader 2. Pathologically confirmed ENE negativity was observed in 117 patients, with an MR-reported ENE negativity in 83 patients for reader 1 and 81 patients for reader 2. The interreader agreement between the two radiologists for assessing ENE was good, with a kappa value of 0.780 (95% CI = 0.671–0.874). The correlation of MR-reported ENE with pathologic findings was confirmed with kappa, sensitivity, and specificity values of 0.233, 52.9%, and 71.5%, respectively. Therefore, the association of MR-reported ENE with pathologic findings showed poor consistency.

### Feature selection, development, and validation of prediction models

**Fig. 3:**
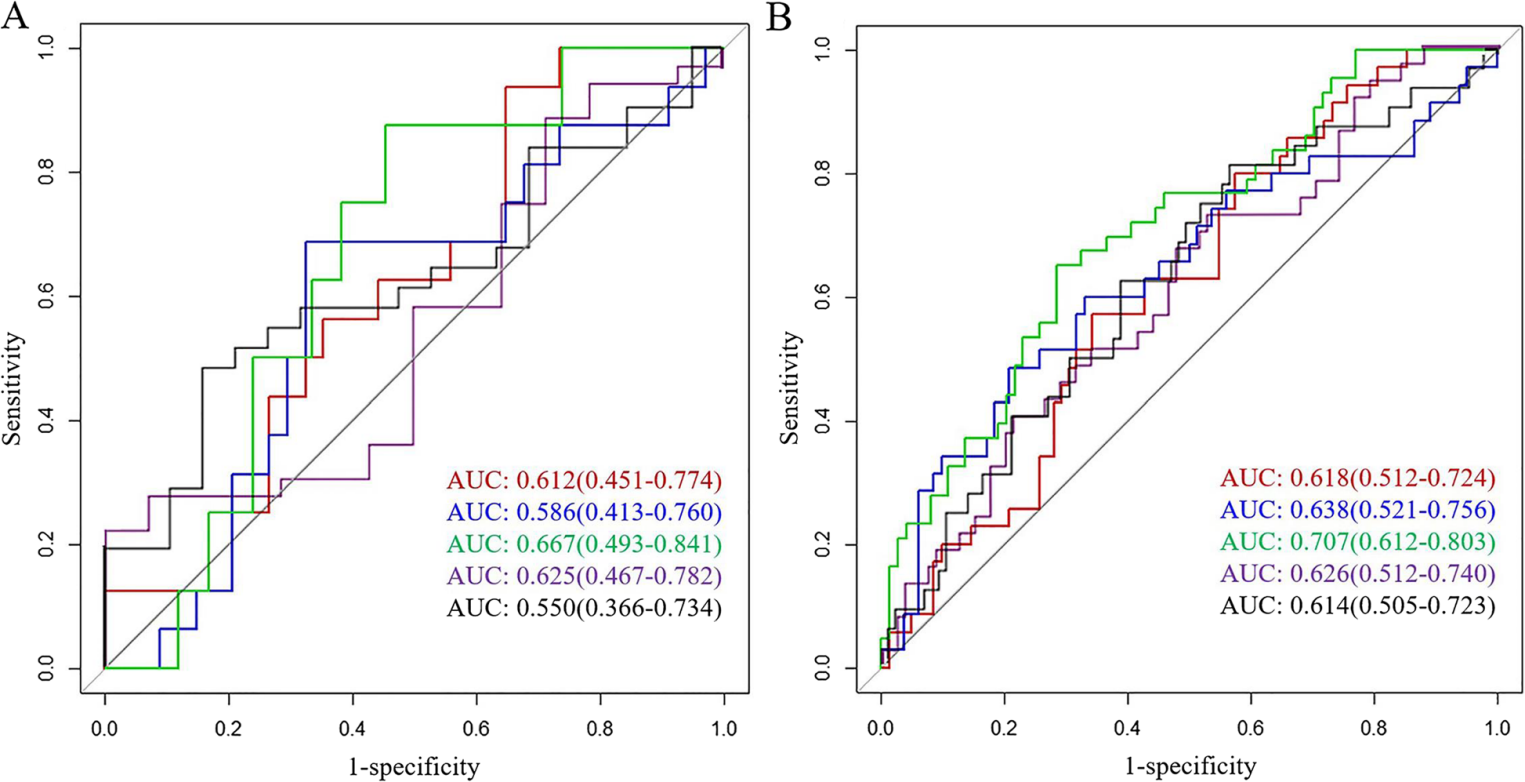
Receiver operating characteristic curves of intratumoral radiomics score (red line), intratumoral&peritumoral-MRF radiomics score (blue line), intratumoral and peritumoral-3-mm radiomics score (green line), peritumoral-3mm radiomics score (purple line), and peritumoral-MRF radiomics score (black line) for predicting extranodal extension in the training cohort (**A**) and validation cohort (**B**)

**Fig. 4:**
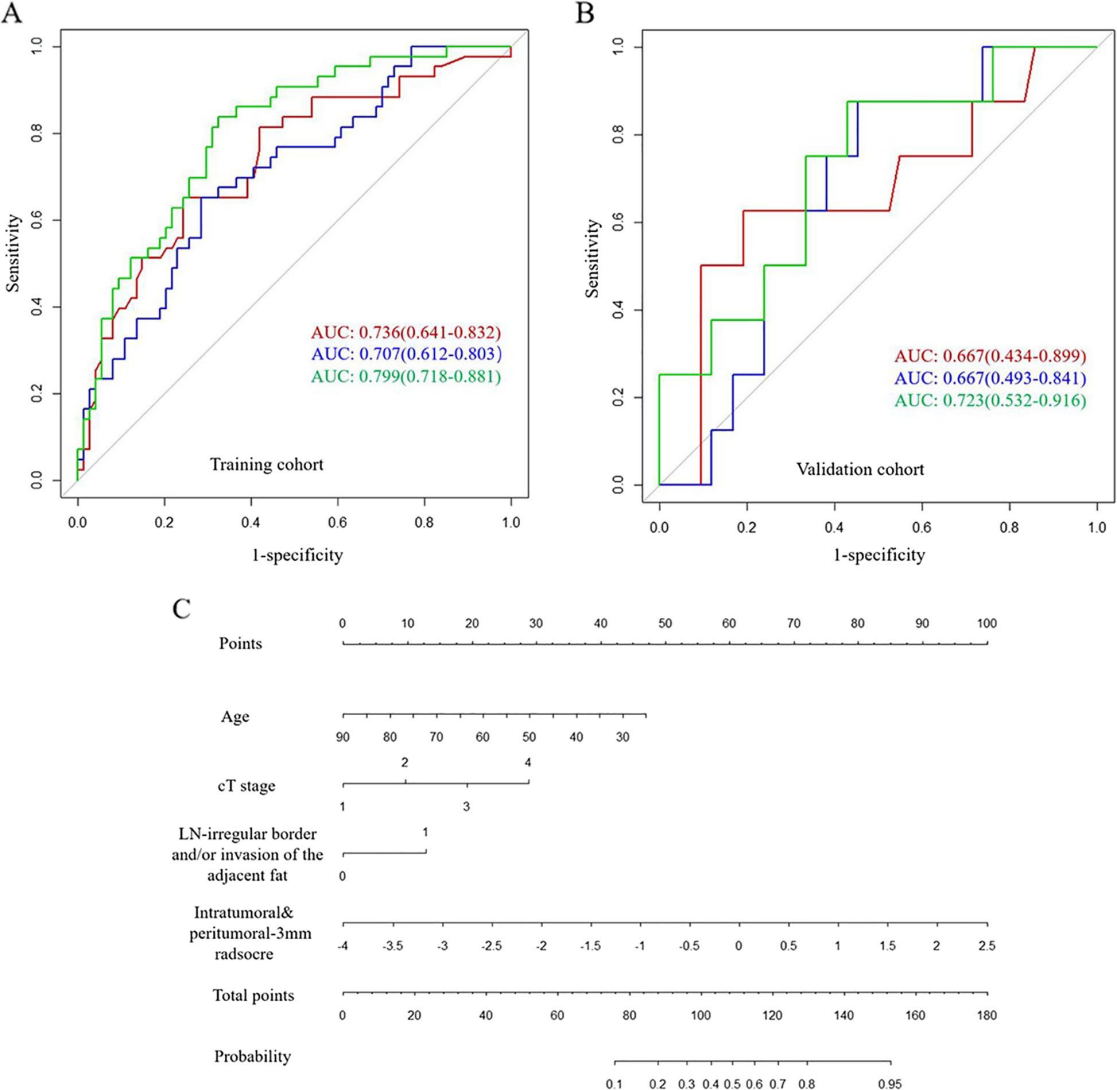
The performance and validation of the final selected model to predict extranodal extension (ENE). ROC of clinical model (red line), intratumoral and peritumoral-3-mm radiomics model (blue line), and nomogram (green line)  for predicting ENE in the training cohort (**A**) and validation cohort (**B**). **C** The predictive nomogram of ENE

**Fig. 5:**
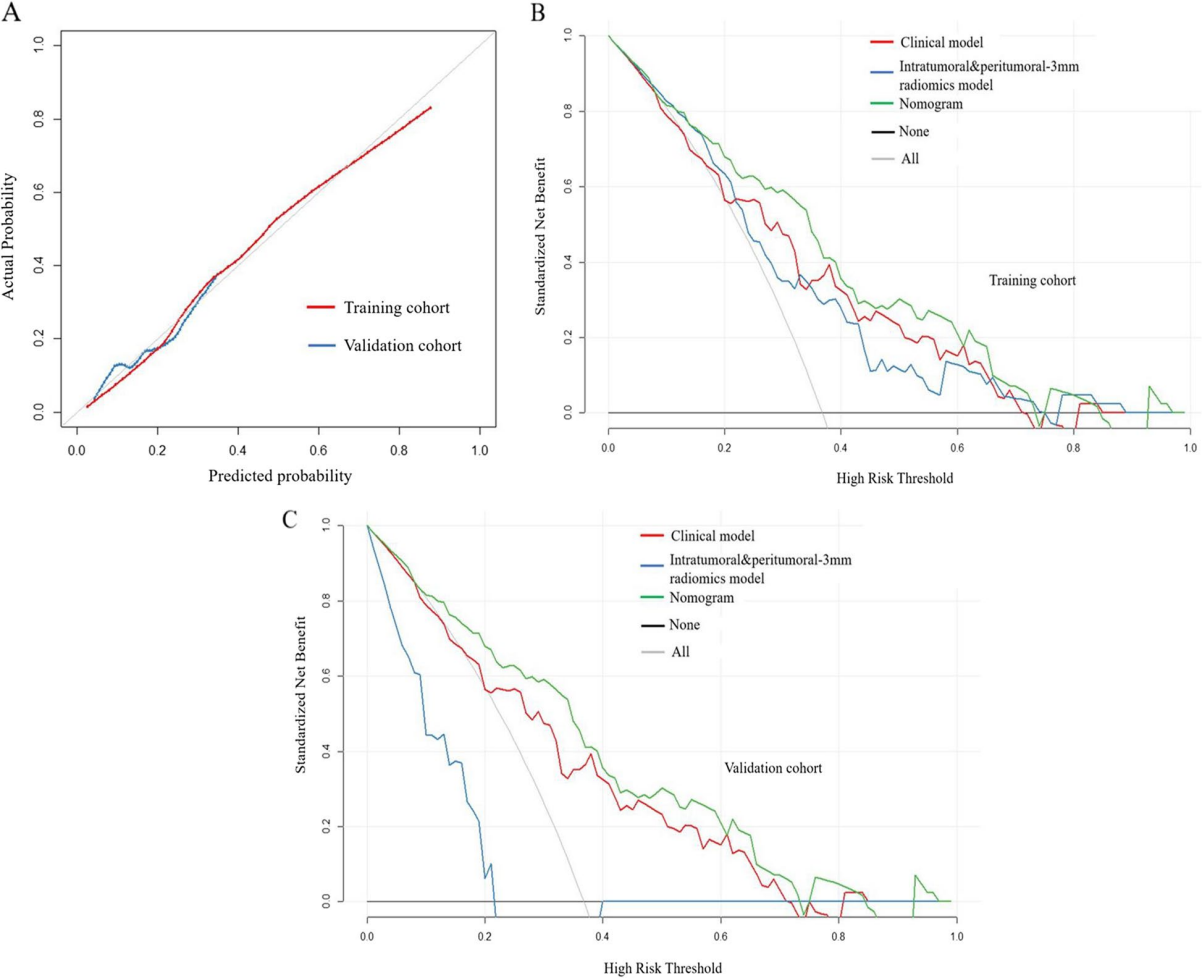
Fit and usefulness evaluation of the clinical-radiomics nomogram. Calibration curve of the clinical-radiomics nomogram for predicting extranodal extension (ENE) in the training cohort (red line) and validation cohort (blue line) (**A**); decision curve analysis (DCA) of the nomogram for assessing its clinical usefulness; this indicates that a nomogram to predict ENE gains more benefit than the “treat all,” “treat none,” radiomics model and the clinical model when the threshold probability ranges from 0.18 to 0.73 in the training cohort (**B**) and from 0.10 to 0.74 in the validation cohort (**C**)

The final formula of the Radscore used to predict ENE is shown in Table S2. Combining intratumoral and peritumoral 3 mm Radscore resulted in the highest capability for predicting ENE, with AUCs of 0.707 and 0.667 in the training cohort and validation cohort, respectively (Fig. 3). A nomogram was constructed by adding the combined intratumoral and peritumoral 3 mm Radscore (odds ratio (OR) = 2.89) to the clinical model (age (OR = 0.95), cT stage (OR = 1.98), and LN-irregular border and/or adjacent fat invasion (OR = 3.36)) as summarized in Table 2. Compared with the clinical model, the nomogram (cutoff, -0.405) provided a slightly higher AUC in the training cohort (0.799 vs. 0.736, *p* = 0.072) and validation cohort (0.723 vs. 0.667, *p* = 0.4) (Tables 3 and 4; Fig. 4). For the nomogram, good agreement between the predicted probability and actual observed probability was demonstrated by the calibration curve. The result of the decision curve indicated that the nomogram had more benefits than the other models for predicting ENE when the threshold probability ranged from 0.18 to 0.73 in the training cohort and from 0.10 to 0.74 in the validation cohort (Fig. 5).

**Table 2:** Univariate and multivariate logistic regression analysis for clinical characteristics and Radscore

Parameters	Univariate analysis		*p* value	Multivariate analysis		*p* value
	OR	95% CI		OR	95% CI	
Intra-peritumoral 3-mm Radscore	2.72	(1.56, 4.73)	< 0.001	2.89	(1.50, 5.56)	< 0.001
Gender	1.31	(0.56, 3.05)	0.582			
Age	0.96	(0.93, 0.99)	0.025	0.95	(0.92, 0.99)	0.017
Location	0.73	(0.42, 1.27)	0.264			
cT-stage	2.03	(1.18, 3.49)	0.01	1.98	(1.02, 3.87)	0.037
Pathological LN	1.23	(0.45–2.88)	0.991			
MRI-reported EMVI	1.30	(0.52, 3.25)	0.576			
CA199	1.00	(0.99, 1.00)	0.819			
CEA	1.00	(0.99, 1.02)	0.347			
Tumor-length	1.00	(0.98, 1.03)	0.487			
Wall thickness	0.98	(0.89, 1.09)	0.742			
LN-irregular border or invasion of the adjacent fat	3.33	(1.5, 7.37)	0.003	3.36	(0.82, 13.73)	0.028
LN-CSE	2.51	(1.16, 5.43)	0.019			
LN-heterogeneous intensity	2.00	(0.91, 4.38)	0.084			

*Radscore* radiomics signature score, *LN* lymph node, *CSE* chemical shift effect, *EMVI* extramural vascular invasion

**Table 3:** Diagnostic performance of the models in the training cohort and validation cohort

Data set	Model	AUC (95% CI)	Sensitivity	Specificity
Training cohort	Intratumoral	0.618 (0.512–0.724)	0.659	0.571
	Intra-Peritumoral-MRF	0.638 (0.521–0.756)	0.793	0.486
	Intra-Peritumoral-3 mm	0.707 (0.612–0.803)	0.716	0.651
	Peritumoral 3 mm	0.626 (0.512–0.74)	0.435	0.812
	Peritumoral-MRF	0.614 (0.505–0.723)	0.475	0.730
	Clinical model	0.736 (0.641–0.832)	0.757	0.651
	Nomogram	0.799 (0.718–0.881)	0.676	0.837
Validation cohort	Intratumoral	0.612 (0.451–0.774)	0.382	0.688
	Intra-Peritumoral-MRF	0.586 (0.413–0.760)	0.676	0.625
	Intra-Peritumoral-3 mm	0.667 (0.493–0.841)	0.619	0.750
	Peritumoral 3 mm	0.625 (0.467–0.782)	0.645	0.421
	Peritumoral-MRF	0.550 (0.366–0.734)	0.75	0.286
	Clinical model	0.667 (0.434–0.899)	0.762	0.625
	Nomogram	0.723 (0.532–0.916)	0.548	0.875

*MRF* mesorectal fat, *Intra-Peritumoral-MRF* combined intratumoral with peritumoral-MRF, *Intra-Peritumoral-3 mm* combined intratumoral and peritumoral 3 mm

**Table 4:** Comparison of the diagnostic performance of the models in the training cohort and validation cohort

Data set	Models comparison	AUC	*p*
Training cohort	Intratumoral vs Intra-Peritumoral-MRF	0.618 vs 0.638	0.8
	Intratumoral vs Intra-Peritumoral-3 mm	0.618 vs 0.707	0.2
	Intratumoral vs clinical model	0.618 vs 0.736	0.1
	Intratumoral vs nomogram	0.618 vs 0.799	0.008
	Intra-Peritumoral-MRF vs Intra-Peritumoral-3 mm	0.638 vs 0.707	0.4
	Intra-Peritumoral-MRF vs clinical model	0.638 vs 0.736	0.2
	Intra-Peritumoral-MRF vs nomogram	0.638 vs 0.799	0.03
	Intra-Peritumoral-3 mm vs clinical model	0.707 vs 0.736	0.7
	Intra-Peritumoral-3 mm vs nomogram	0.707 vs 0.799	0.03
	Clinical model vs nomogram	0.736 vs 0.799	0.072
Validation cohort	Intratumoral vs Intra-Peritumoral-MRF	0.612 vs 0.586	0.8
	Intratumoral vs Intra-Peritumoral-3 mm	0.612 vs 0.667	0.3
	Intratumoral vs clinical model	0.612 vs 0.667	0.7
	Intratumoral vs nomogram	0.612 vs 0.723	0.4
	Intra-Peritumoral-MRF vs Intra-Peritumoral-3 mm	0.586 vs 0.667	0.2
	Intra-Peritumoral-MRF vs clinical model	0.586 vs 0.667	0.7
	Intra-Peritumoral-MRF vs nomogram	0.586 vs 0.723	0.4
	Intra-Peritumoral-3 mm vs clinical model	0.667 vs 0.667	0.8
	Intra-Peritumoral-3 mm vs nomogram	0.667 vs 0.723	0.9
	Clinical model vs nomogram	0.667 vs 0.723	0.4

*MRF* mesorectal fat, *Intra-Peritumoral-MRF* combined intratumoral with peritumoral-MRF, *Intra-Peritumoral-3 mm* combined intratumoral and peritumoral 3 mm

### Subgroup analyses

Subgroup analyses of the three models are shown in Fig. S2 and Table S3. There were 3 ENE + patients and 26 ENE- patients at the T1-T2 stage. For differentiating ENE + from ENE- patients at the T1-T2 stage, both the clinical model and the nomogram had similar AUCs, which were slightly higher than that of the radiomics model (0.705 vs. 0.660, *p* = 0.076, i.e., not statistically significant). There were 48 ENE + patients and 90 ENE-patients at the T3a/b-T4a stage. For differentiating ENE + from ENE- patients at the T3a/b-T4a stage, the nomogram showed better AUCs than the clinical model (0.725 vs. 0.656, *p* = 0.041) and slightly higher AUCs than the radiomics model (0.725 vs. 0.697, *p* = 0.085, i.e., not statistically significant).

### Survival analysis

**Fig. 6:**
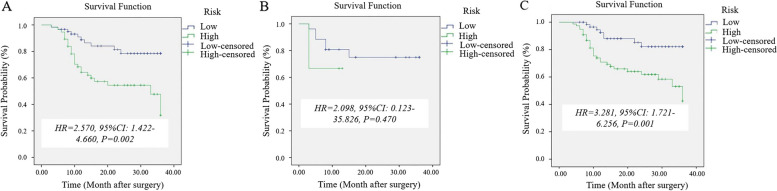
Kaplan–Meier survival curves of the nomogram-based extranodal extension (ENE) for 3-year recurrence-free survival in patients with rectal cancer in the entire cohort (**A**), at T1-T2 stage (**B**), and T3a/b-T4a stage (**C**)

The median follow-up of the event-free patients was 24 months (range, 6–36 months) and 22 months (range, 7–36 months) in the training cohort and validation cohort, respectively. The 51 patients with ENE had a higher rate of recurrence than the 116 patients without ENE (43.1% vs. 18.9%). In the training cohort, there were 35 patients (35/117, 29.9%) with locoregional or distant relapse after a median duration of 10 months (3–36 months). In the validation cohort, there were 9 patients (9/50, 18%) with locoregional or distant relapse after a median duration of 8 months (4–29 months). As shown in Kaplan–Meier survival curves (Fig. 6), patients with low clinical-radiomics nomogram score-based ENE- (≤ -0.405) showed better 3-year RFS than those with high score-based ENE + (> -0.405).

Univariate and multivariate Cox analyses showed that EMVI (hazard ratio [HR] = 2.523, 95% CI = 1.247–5.106, *p* = 0.010) and clinical-radiomics nomogram-based ENE (HR = 2.625, 95% CI = 1.233–5.586, *p* = 0.012) were independent risk factors for 3-year RFS (Table 5). EMVI and clinical-radiomics nomogram-based ENE assessments were performed to construct the prognostic model for 3-year RFS and indicated good performance, with AUCs of 0.761 in the training cohort and 0.710 in the validation cohort.

**Table 5:** The results of univariate and multivariate Cox regression analyses for assessing 3-year recurrence-free survival in the training cohort

Variate	Univariate Cox		Multivariate Cox	
	*p*	HR (95% CI)	*p*	HR (95% CI)
Sex (male)	0.033	2.805 (1.086–7.248)	0.180	1.966 (0.732–5.281)
Age (> 65)	0.189	1.569 (0.801–3.070)		
Treatment (surgery plus postoperative adjuvant therapy)	0.136	2.213 (0.779–6.283)		
T-stage (T3a/b-4a)	0.578	1.310 (0.506–3.395)		
Pathological LNM ( + )	0.027	2.917 (1.129–7.536)	0.308	1.771 (0.591–5.307)
MRI-reported EMVI ( + )	0.001	3.162 (1.589–6.292)	0.010	2.523 (1.247–5.106)
CA199 (> 37)	0.584	1.341 (0.469–3.833)		
CEA (> 6)	0.079	1.817 (0.932–3.543)	0.370	1.378 (0.684–2.776)
Tumor length (> 42.5 mm)	0.466	1.283 (0.656–2.508)		
Wall thickness (> 12 mm)	0.237	0.669 (0.344–1.302)		
LN-irregular border and/or adjacent fat invasion ( + )	0.057	1.908 (0.982–3.709)	0.193	0.531 (0.204–1.377)
LN-chemical shift effect ( + )	0.081	1.816 (0.929–3.555)	0.391	1.603 (0.545–4.716)
LN-heterogeneous intensity ( + )	0.154	1.629 (0.833–3.185)		
Intratumoral Radscore (> -0.756)	0.797	1.091 (0.561–2.122)		
Intratumoral + peritumoral-MRF Radscore (> -0.930)	0.911	0.963 (0.496–1.869)		
Intratumoral + peritumoral 3-mm Radscore (> -0.531)	0.289	1.451 (0.730–2.886)		
Clinical-radiomics combined model score based-ENE + (> -0.405)	0.003	3.099 (1.485–6.466)	0.012	2.625 (1.233–5.586)

Sex (female), age (≤ 65), treatment (surgery only), T-stage (T1-2), lymph node metastasis (LNM) (-), extramural vascular invasion (EMVI) (-), CA199 (≤ 37), CEA (≤ 6), tumor length (≤ 42.5 mm), wall thickness (≤ 12 mm), LN-irregular border (-), LN-chemical shift effect (-), LN-heterogeneous intensity (-), intratumoral Radscore (≤  -0.756), intra- and peritumoral-mesorectal fat (MRF) Radscore (≤ -0.930), intra- and peritumoral 3-mm Radscore (≤ -0.531), and clinical-radiomics combined model score-based ENE + (≤ -0.405) were as a reference in univariate and multivariate COX analysis

## Discussion

From several studies, scholars have reported that intratumoral radiomics signatures could predict LNM and tumor deposits in rectal cancer [17, 30, 31]. In this study, we also assessed ENE with radiomic signatures from the primary tumor, instead of lymph nodes. The explanation could be that it was sometimes difficult to completely maintain the node-to-node correspondence between surgical histology and MR-identified nodes. Moreover, small LNs (e.g., < 5 mm) may have positive ENE, but were very difficult for the radiologist to draw ROI on given their small size. Even though small lymph nodes could be identified by the radiologist, drawing the entire lymph node on MR images with a slice thickness of 3 mm was also difficult. Since the risk of metastases is fundamentally driven by the primary tumor, we can hypothesize that radiomics analysis of the primary tumor may help identify the status of ENE. We found that T2WI-based radiomics from the intratumoral region could predict ENE with an AUC of 0.612. Jin et al. and Chen et al. reported that radiomics features obtained from intratumoral and peritumoral fat were used to construct a model for predicting tumor deposits [16, 32]. These studies found that the combined model incorporating intratumoral and peritumoral fat and clinical factors provided good performance for predicting tumor deposits. However, the radiomic features in these studies were extracted from ultrasound or CT images, which were not the best examination modality for rectal cancer. Jayaprakasam et al. reported that MRI radiomics features from MRF showed good performance for predicting tumor recurrence and response to neoadjuvant chemoradiation therapy in rectal cancer [33]. A recent study also showed that combining intratumoral and MRF radiomics models provided better performance than a single intratumoral radiomics model for predicting tumor deposits [34]. These studies defined the peritumoral region by drawing along the mesorectal fascia. However, some studies have defined the peritumoral region as the area immediately surrounding the tumor [20, 26, 35–37]. Therefore, it is uncertain whether we should combine intratumoral and MRF radiomics features or intratumoral and peritumoral regions immediately surrounding the tumor. In this study, we compared different regional Radscores and found that the combined intratumoral and peritumoral 3 mm Radscore achieved the highest capability for predicting ENE, with an AUC of 0.723. For radiomics features from the intratumoral region, the negative coefficient (-0.592) of Zone Percentage indicated a coarser texture, implying notable tumor heterogeneity. For peritumoral 3 mm radiomics features, the positive coefficient of gray-level variance indicating the variance in gray-level intensity for the runs implies tumor heterogeneity. For peritumoral-MRF radiomics features, the positive coefficient of short-run high gray-level emphasis and IDN imply homogeneous textures [38]. These findings may suggest that the peritumoral region far from the tumor contains less information than the region immediately surrounding the tumor. The probable interpretation could be related to the peritumoral immune microenvironment, which was mainly in the region immediately adjacent to the tumor [39]. Moreover, in a previous study, it was reported that tumor cells were separated from the infiltrating edge of the tumor and migrated to the surrounding stroma, indicating that tumor-budding cell clusters mainly existed at the edge of the tumor [40]. These findings may indicate that the region adjacent to the tumor plays an important role in metastatic LN.

To our knowledge, no studies have used clinical factors to select noninvasive independent predictors of ENE. We found that age, cT-stage, and LN-irregular border and/or invasion of the adjacent fat on MRI were independent predictors for ENE. Heterogeneous LN intensity and disappearance of the CSE were not independent risk factors for ENE. The most likely explanation is that heterogeneous intensity in LNs and the disappearance of LN-CSE are not unique to ENE, which can be seen in most metastatic LNs. Moreover, tumor cells inside the subcapsular sinus that do not break through the LN capsule may also influence the uniform fat-water interface and thus destroy the normal chemical shift effect [23]. In this study, LN-irregular border and/or adjacent fat invasion on MRI was considered a morphological feature for assessing ENE. We found that the correlations of LN-irregular border and/or adjacent fat invasion with pathologic findings were validated with kappa, sensitivity, and specificity values of 0.233, 52.9%, and 71.5%, respectively. The explanation could be that it was sometimes difficult to assess the border status of small nodes because of the restriction of spatial resolution. Moreover, there is a limitation for current imaging modalities to accurately identify microscopic ENE [41, 42]. Therefore, we may conclude that it was difficult to assess ENE using morphological features on MRI. Our findings indicated that adding the combined intratumoral and peritumoral 3 mm Radscore to the clinical model could improve the benefit compared with the clinical model alone for the assessment of ENE. However, there was no statistical significance for AUC between the clinical model and the nomogram. A possible explanation could be the small sample size of patients and lower incidence of ENE + in this study. Moreover, the proportions of patients with and without ENE greatly differed between the training cohort and the validation cohort. Therefore, further studies with larger sample sizes should be performed to confirm our findings.

For the subgroup analyses, our results showed that both the clinical model and clinical-radiomics nomogram had similar AUCs of 0.705 for differentiating ENE + from ENE- patients at the T1-T2 stage. The explanation could be that the incidence of ENE + is low (10.3%, 3/29) at the T1-T2 stage. For differentiating ENE + from ENE- patients at the T3a/b-T4a stage, the clinical-radiomics nomogram performed significantly better than the clinical model (AUC, 0.725 vs. 0.656). The explanation could be that the incidence of ENE + is high (34.8%, 48/138) at the T3a/b-T4a stage. Moreover, greater heterogeneity exists in T3a/b-T4a stage rectal cancers than in T1-T2 stage cancers, and these features cause substantial differences in radiomic features [43]. Therefore, combining these radiomics features with clinical risk factors may lead to a better prediction of ENE than the clinical model alone. These results may indicate that the clinical-radiomics nomogram can predict ENE stratified by tumor T staging. In addition, patients with ENE had a higher rate of recurrence than patients without ENE (51.9% vs. 8.3%). Our study showed that patients with low clinical-radiomics combined model score-based ENE had better 3-year RFS than patients with high scores. Multivariate Cox analysis showed that clinical-radiomics nomogram-based ENE in addition to MRI-reported EMVI were independent risk factors for predicting 3-year RFS. A previous study also confirmed that MRI-reported EMVI was strongly associated with distant recurrence [44]. Our findings may lead to the conclusion that T stage and N stage are not sufficient for classifying the patient, and it may be more sensible to include additional indicators, such as in the clinical-radiomics nomogram-based ENE and EMVI.

There are some limitations in this study. Firstly, due to the low incidence of ENE, the sample size of patients with ENE enrolled in this study was small. To prevent the model from being affected by data bias during the training process, relatively more positive samples were randomly allocated to the training set data to ensure that the ratio of ENE negative and positive in the training set data was within the range of 2:1. Although this allocation scheme would make the proportion of patients with ENE negative and positive very different in the validation cohort, the incidence rate of ENE was approximately 22% [45], which would not affect practical clinical applications. Secondly, this is a single-center study, and the model will need to be confirmed with external validation data. Thirdly, this study only included T2WI-based radiomics analysis. Other MRI sequences, such as DWI, were excluded. Fourthly, although we endeavored to keep the LNs on preoperative images matched with histopathologic results, it was sometimes difficult to completely maintain the node-to-node correspondence. However, clinicians generally focus only on the presence or absence of ENE in patients with rectal cancer. Therefore, in this study, the analysis was performed on a per-patient basis for ENE rather than on a per-node basis. Finally, patients with T3a/b-T4a or N + rectal cancer did not receive preoperative chemoradiotherapy. In this study, we found that the 3-year RFS rate was 73.5%, which did not differ when compared with standard therapy of preoperative chemoradiotherapy, surgery, and postoperative adjuvant chemotherapy with 3-year RFS rate of 75% [46]. This finding seems to indicate that preoperative chemoradiotherapy has not been shown to significantly increase the 3-year RFS rate compared with surgery alone [47].

In summary, our study provides a clinical-radiomics nomogram that combines intratumoral and peritumoral 3 mm Radscore, age, cT stage, and LN-irregular border and/or adjacent fat invasion for the preoperative prediction of ENE. Combining this nomogram-based ENE prediction with EMVI could help identify patients at high risk for recurrence and promote personalized treatment.

### Supplementary Information

**Additional file 1: Figure S1.** Representative examples of evaluating extranodal extension (ENE) on T2WI and histopathology. **Figure S2.** Receiver operating characteristic curves of intratumoral & peritumoral-3 mm radiomics model, clinical model, and the clinical-radiomics nomogram for identifying extranodal extension at T1-T2 stage (A) and T3a/b-T4a stage (B) of rectal cancer. **Table S1.** The correlation between MR-reported extranodal extension (ENE) and pathological results. **Table S2.** Radiomics features score (radscore) formula of different models for predicting extranodal extension. **Table S3.** Subgroup analysis of the radiomics model, clinical model, and the clinical-radiomic nomogram.

## Data Availability

The datasets generated during and analyzed during the current study are not publicly available due to PACS system regulated by Sichuan Provincial People’s Hospital but are available from the corresponding author upon reasonable request.

## Abbreviations

AJCC: American Joint Committee on Cancer
AUC: Areas under the receiver operating characteristic curve
CA199: Carbohydrate antigen 199
CEA: Carcinoembryonic antigen
CI: Confidence interval
CSE: Chemical shift effect
DCA: Decision curve analysis
EMVI: Extramural vascular invasion
ENE: Extranodal extension
FOV: Field of view
HR: Hazard ratio
ICC: Intraclass correlation coefficient
LN: Lymph node
LNM: Lymph node metastasis
MRF: Mesorectal fat
OR: Odds ratio
RFS: Recurrence-free survival
ROC: Receiver operating characteristic
T2WI: T2-weighted images
