# Supplementary material for: T2WI-based MRI radiomics for the prediction of preoperative extranodal extension and prognosis in resectable rectal cancer
Source: Insights Imaging. 2024 Feb 27;15:57. doi: 10.1186/s13244-024-01625-8 (PMC10899552; doi:10.1186/s13244-024-01625-8)
Supplement: Supplementary file 1 — Additional file 1: Figure S1. Representative examples of evaluating extranodal extension (ENE) on T2WI and histopathology. Figure S2. Receiver operating characteristic curves of intratumoral & peritumoral-3 mm radiomics model, clinical model, and the clinical-radiomics nomogram for identifying extranodal extension at T1-T2 stage (A) and T3a/b-T4a stage (B) of rectal cancer. Table S1. The correlation between MR-reported extranodal extension (ENE) and pathological results. Table S2. Radiomics features score (radscore) formula of different models for predicting extranodal extension. Table S3. Subgroup analysis of the radiomics model, clinical model, and the clinical-radiomic nomogram. [file 13244_2024_1625_MOESM1_ESM.pdf]

T2WI-based MRI radiomics for the prediction of preoperative extranodal extension and prognosis in resectable rectal cancer

ELECTRONIC SUPPLEMENTARY MATERIAL

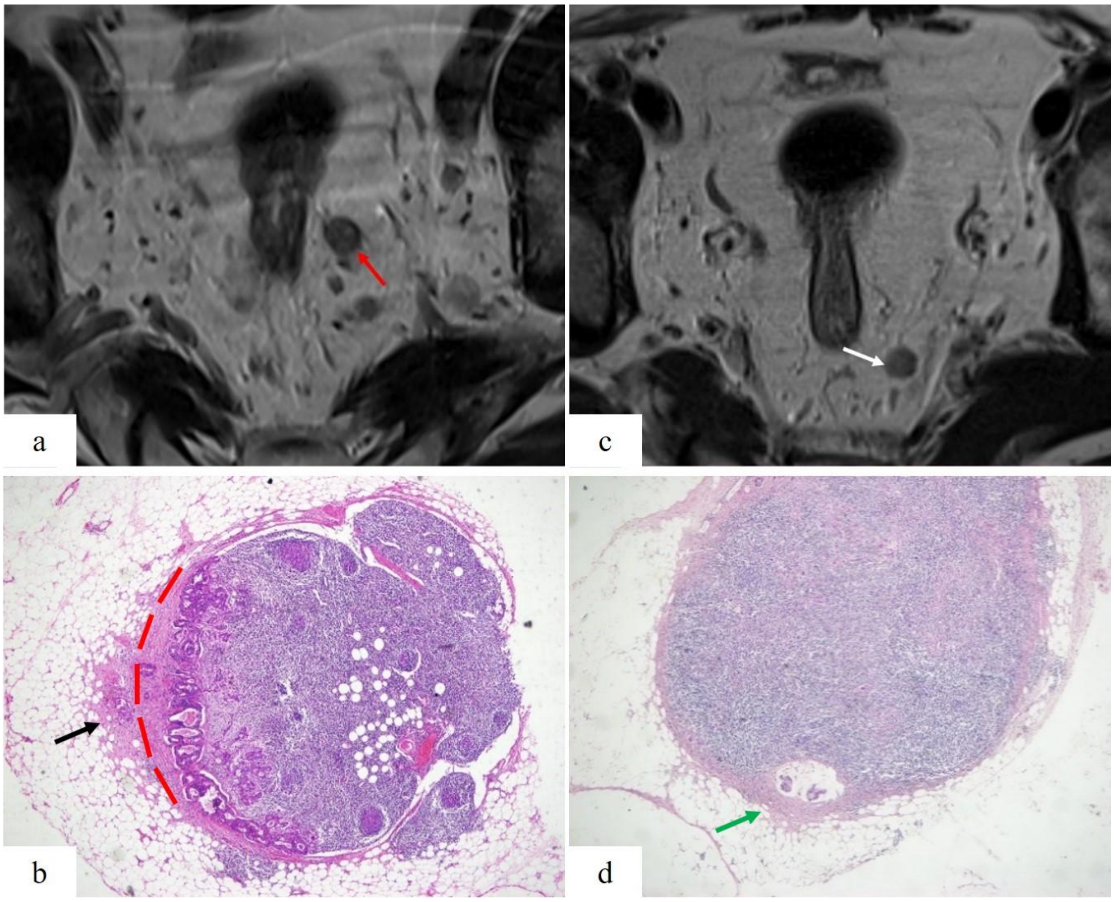

Figure S1

Representative examples of evaluating extranodal extension (ENE) on T2WI and histopathology. **a-b** T2-weighted MR image shows irregularity of the lymph node (LN) margin, interrupted low signal of LN capsule, and invasion of the adjacent fat (red arrow). A histopathologically (Original magnification 40 ×; hematoxylin-eosin stains) proved ENE positive, with tumor cells breaking through the capsule of LN (dashed line) into the surrounding perinodal adipose tissues (black arrow). **c-d** T2-weighted MR image shows a smooth and regular LN margin (white arrow). A histopathologically

(Original magnification 40 ×; hematoxylin-eosin stains) proved ENE negative, with tumor cells confined to the capsule of LN (green arrow).

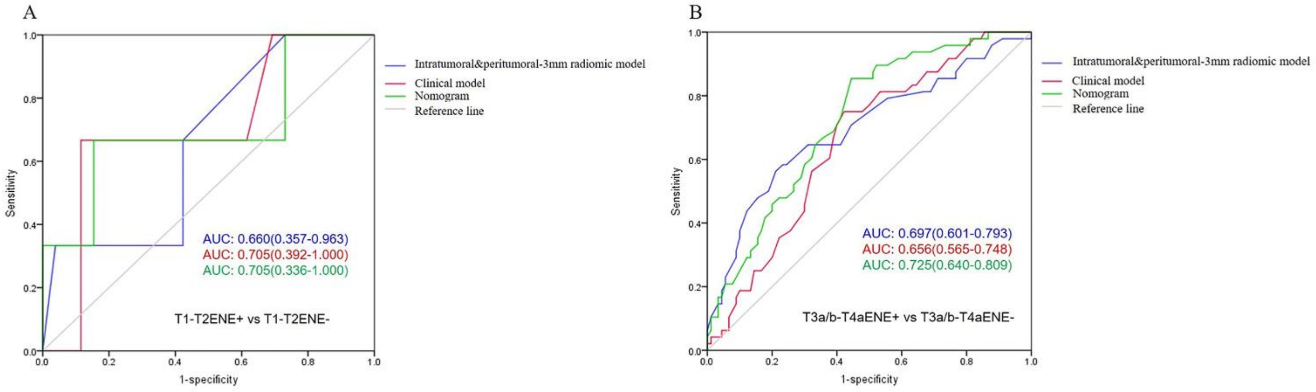

Figure S2

Receiver operating characteristic curves of intratumoral & peritumoral-3 mm radiomics model, clinical model, and the clinical-radiomics nomogram for identifying extranodal extension at T1-T2 stage (A) and T3a/b-T4a stage (B) of rectal cancer.

Table S1 The correlation between MR-reported extranodal extension (ENE) and pathological results.

Variants	Pathological-ENE positive (n=51)	Pathological-ENE negative (n=116)	*P*-value
Reader 1			0.003
MR-reported ENE negative	24(47.06%)	83(71.55%)	
MR-reported ENE positive	27(52.94%)	33(28.45%)	
Reader 2			0.010
MR-reported ENE negative	25(49.02%)	81(69.83%)	
MR-reported ENE positive	26(50.98%)	35(30.17%)	

Table S2 Radiomics features score (radscore) formula of different models for predicting extranodal extension.

Models	Radscore formula
Intratumoral radscore	-0.592*Intra_log.sigma.3.0.mm.3D_glszm_ZonePercentage - 0.945
Intratumoral+peritumoral-MRF radscore	0.433*Pri.MRF_wavelet.HLL_gldm_SmallDependenceLowGrayLevelEmphasis-0.86
Intratumoral+peritumoral-3mm radscore	-0.595*Intra_log.sigma.3.0.mm.3D_glszm_ZonePercentage +0.536*Pri.3mm_original_firstorder_interquartileRange+ 0.644*Intra_log.sigma.3.0.mm.3D_glcm_Imc1 - 0.802*Pri.3mm_original_firstorder_Kurtosis + 0.779*Pri.3mm_log.sigma.2.0.mm.3D_glrlm_LongRunEmphasis-0.61
Peritumoral-MRF radscore	0.999*Pri.3mm_lbp.3D.k_glrlm_ShortRunHighGrayLevelEmphasis+0.905*Pri.3mm_lbp.3D.k_glcm_Idn-0.747
Peritumoral-3mm radscore	0.422*Pri.MRF_lbp.3D.k_glrlm_GrayLevelVariance-1.003

Note: MRF, mesorectal fat

Table S3 Subgroup analysis of the radiomics model, clinical model, and the clinical-radiomic nomogram

Model	Subgroup	AUC (95%CI)	Sensitivity	Specificity
Clinical model	T1-T2 stage	0.705(0.392-1.000)	66.7%	88.5%
	T3-T4 stage	0.656 (0.565-0.748)	70.8%	60.0%
Intratumoral & Peritumoral-3mm radiomics model	T1-T2 stage	0.660(0.357-0.963)	66.7%	57.7%
	T3-T4 stage	0.697(0.601-0.793)	64.6%	68.9%
Nomogram	T1-T2 stage	0.705(0.336-1.000)	66.7%	84.6%
	T3-T4 stage	0.725(0.640-0.809)	85.4%	56.0%
